# A machine learning model for visualization and dynamic clinical prediction of stroke recurrence in acute ischemic stroke patients: A real-world retrospective study

**DOI:** 10.3389/fnins.2023.1130831

**Published:** 2023-03-27

**Authors:** Kai Wang, Qianqian Shi, Chao Sun, Wencai Liu, Vicky Yau, Chan Xu, Haiyan Liu, Chenyu Sun, Chengliang Yin, Xiu’e Wei, Wenle Li, Liangqun Rong

**Affiliations:** ^1^Department of Neurology, The Second Affiliated Hospital of Xuzhou Medical University, Xuzhou, Jiangsu, China; ^2^Key Laboratory of Neurological Diseases, The Second Affiliated Hospital of Xuzhou Medical University, Xuzhou, Jiangsu, China; ^3^State Key Laboratory of Molecular Vaccinology and Molecular Diagnostics & Center for Molecular Imaging and Translational Medicine, School of Public Health, Xiamen University, Xiamen, China; ^4^Department of Neurosurgery, The Second Affiliated Hospital of Soochow University, Suzhou, China; ^5^Department of Orthopaedic Surgery, The First Affiliated Hospital of Nanchang University, Nanchang, China; ^6^Division of Oral and Maxillofacial Surgery, Columbia University Irving Medical Center, New York, NY, United States; ^7^Department of Dermatology, Xianyang Central Hospital, Xianyang, China; ^8^Faculty of Medicine, Macau University of Science and Technology, Macau, China

**Keywords:** stroke, recurrence, machine learning, SHAP, web calculator

## Abstract

**Background and purpose:**

Recurrent stroke accounts for 25–30% of all preventable strokes, and this study was conducted to establish a machine learning-based clinical predictive rice idol for predicting stroke recurrence within 1 year in patients with acute ischemic stroke (AIS).

**Methods:**

A total of 645 AIS patients at The Second Affiliated Hospital of Xuzhou Medical University were screened, included and followed up for 1 year for comprehensive clinical data. Univariate and multivariate logistic regression (LR) were used to screen the risk factors of stroke recurrence. The data set was randomly divided into training set and test set according to the ratio of 7:3, and the following six prediction models were established by machine algorithm: random forest (RF), Naive Bayes model (NBC), decision tree (DT), extreme gradient boosting (XGB), gradient boosting machine (GBM) and LR. The model with the strongest prediction performance was selected by 10-fold cross-validation and receiver operating characteristic (ROC) curves, and the models were investigated for interpretability by SHAP. Finally, the models were constructed to be visualized using a web calculator.

**Results:**

Logistic regression analysis showed that right hemisphere, homocysteine (HCY), C-reactive protein (CRP), and stroke severity (SS) were independent risk factors for the development of stroke recurrence in AIS patients. In 10-fold cross-validation, area under curve (AUC) ranked from 0.777 to 0.959. In ROC curve analysis, AUC ranged from 0.887 to 0.946. RF model has the best ability to predict stroke recurrence, and HCY has the largest contribution to the model. A web-based calculator https://mlmedicine-re-stroke2-re-stroke2-baylee.streamlitapp.com/ has been developed accordingly.

**Conclusion:**

This study identified four independent risk factors affecting recurrence within 1 year in stroke patients, and the constructed RF-based prediction model had good performance.

## 1. Introduction

Stroke is characterized by acute focal injury of the central nervous system (i.e., brain, retina, or spinal cord) resulting in neurological dysfunction due to sudden rupture of blood vessels or obstruction of blood flow. It is categorized into ischemic and hemorrhagic stroke, while the incidence of the former is higher than that of the latter, accounting for 60–70% of all strokes (Sacco et al., 2013). The major clinical manifestation of stroke is the sudden onset of focal neurological deficits, whose clinical diagnosis is further complemented with imaging of the brain and its vascular trees (Campbell and Khatri, 2020). Epidemiological data suggest that stroke is the second leading cause of death and disability worldwide, causing tremendous burden shared by low- and middle-income countries (Saini et al., 2021). As suggested by 2016 global burden of disease data that one in four people will have a stroke in their lifetime (GBD 2016 Neurology Collaborators, 2019), its prevalence is approximately equal in men and women. However, incidence of stoke is higher in older women (more than 50% higher comparing to men aged 75 years or older), and among some races (e.g., 1.91 per 1,000 in Black or African American and 0.88 per 1,000 in Caucasians) (Virani et al., 2020). Data from 2010 to 2017 showed a 5.3% increase in stroke morbidity and mortality and a 19.3% increase in prevalence, respectively (Goldstein, 2020).

Despite effective treatment approaches, stroke patients are still at measurable risk of recurrent episodes after initial recovery. Recurrent strokes account for 25–30% of all preventable strokes, a majority of which are ischemic strokes, and their onset lead to a higher mortality and disability rate than the initial episode (Luengo-Fernandez et al., 2012). The risk rates for early recurrence of ischemic stroke are approximately 5% at 7 days, and 10% at 14 days, respectively; the long-term recurrence risk rates are approximately 11.1% [95% confidence interval (CI) 9–0 to 13.3] at 1 year (Hankey, 2014). Therefore, the identification of risk factors for stroke recurrence is beneficial to identify populations of high-risk recurrence, ensuring early intervention to reduce the morbidity and mortality. Previous studies have shown that pathophysiological factors and lifestyle factors are all influential factors leading to stroke recurrence. In addition, history of previous cerebrovascular events and stroke subtypes are also important risk factors for recurrence (Chin et al., 2018). Therefore, it is crucial to develop predictive models for effective secondary prevention and management.

With rapid development of precision medicine in the recent years, data science and predictive analytics take on significant roles for physicians to deliver individualized care. However, clinical application of models to predict recurrent stroke using regression or other statistical methods is often limited by the narrow range of variables (Chaudhary et al., 2019) as studies have shown that the area under the receiver operating characteristic (ROC) curve for multivariate logistic models developed using clinical and retinal characteristics for recurrent stroke within 1 year is 0.71–0.74 [higher area under curve (AUC) values indicate better model predictive power] (Yuanyuan et al., 2020). Similarly, when machine learning (ML) is used with single- or multi-omics medical data, more details can be mined from the data and better diagnostic and prognostic tools can be developed compared to traditional statistical regression models (Bersanelli et al., 2016; Erickson et al., 2017; Dias-Audibert et al., 2020; Fleuren et al., 2020). Studies have demonstrated that ML can successfully predict favorable outcomes for up to 3 months after acute stroke event and that the area under the curve of deep neural network models is significantly higher than the Astral score (0.888 vs. 0.839; *P* < 0.001) (Heo et al., 2019). In addition, ML can be used to efficiently label stroke patients in the emergency setting to facilitate triage (Abedi et al., 2020), as well as to monitor predictive models for long-term recurrent stroke (5 years) by using six of its algorithmic models (Abedi et al., 2021).

In the current study, we constructed six different prediction models by adding observational indicators and explored factors influencing recurrence in all stroke patients based on 1-year follow-up data, evaluated their performances based on sensitivity, specificity, accuracy, and subject operating characteristic curve (ROC), and analyzed the relative importance and interpretability of different factors on the models. We aimed to provide a reference for identifying stroke patients at high risk of recurrence, which is conducive to early diagnosis, and treatment of stroke recurrence, leading to improved survival and recovery of patients.

## 2. Materials and methods

### 2.1. Data sources, inclusion criteria, exclusion criteria

The data of this study were obtained from patients who were diagnosed with acute ischemic stroke (AIS) at The Second Affiliated Hospital of Xuzhou Medical University from August 2017 to July 2019. The inclusion criteria for AIS patients included: ischemic stroke diagnoses following the World Health Organization criteria and from onset of symptom to hospitalization less than 24 h. Exclusion criteria were as follows: (1) incomplete clinical information. (2) Patients with severe organ dysfunction. (3) Inadequate ancillary tests. (4) Follow-up time for less than 1 year. (5) Patients with disturbance of consciousness and severe aphasia. The study was approved by the Ethics Committee of The Second Affiliated Hospital of Xuzhou Medical University [ethics number: (2020) 081603], and all patients signed a written consent form.

Data of enrolled patients were collected, including demographic data (age, sex); vascular risk factors (hypertension, diabetes, ischemic heart disease); baseline blood pressure [SBP and diastolic blood pressure (DBP)]; trial of org 10 172 in acute stroke treatment (TOAST) (large-artery atherosclerosis, cardioembolism, small-vessel occlusion, acute stroke of other determined etiology, stroke of undetermined etiology); stroke severity (SS) [based on the National Institutes of Health NIHSS, NIHSS score ≤ 8 for mild stroke, NIHSS score ≥ 9 for moderate to severe stroke (Muchada et al., 2014), all assessments completed on admission]; MRI readings [stroke distribution (anterior circulation, posterior circulation, anterior/posterior circulation), hemispheric laterality (left, right, bilateral), number of stroke lesions (single, multiple); stroke lesion site (cortical, cortico-subcortical, subcortical, subcortical, subcortical) (subcortical, subcortical, brainstem and cerebellum)], laboratory tests [total cholesterol, triglycerides, low-density lipoprotein (LDL), fasting blood glucose (FBG), homocysteine (HCY), uric acid (UA), fibrinogen (FIB), myoglobin (MB), C-reactive protein (CRP), d-dimer, brain natriuretic peptide (BNP), HBALC, neuronal specific enolase (NSE), S-100β], and clinical treatment [thrombolysis, thrombectomy, antiplatelet, anticoagulation, statin, pump inhibitor therapy (PPI)]. complications of stroke [dysphagia (Banda et al., 2022), stroke-associated pneumonia (SAP) (Qiu et al., 2022)].

### 2.2. Statistical methods

The collected clinicopathological and biochemical data were subjected to statistical analysis and model construction using R (version4.0.5)^1^ and Python (version3.8). Firstly, based on various types of data, continuous variables were expressed as mean ± standard deviation and compared using an independent samples *t*-test; categorical variables were expressed as frequency (percentage, %) and analyzed with χ2 test. Logistic regression (LR) analysis was used to identify risk factors independently associated with stroke recurrence. Variables with *P*-values less than 0.05 in the results of univariate LR analysis were included in multivariate LR analysis. Finally, factors with a *P*-value < 0.05 in the results of multivariate LR analysis were identified as independent risk factors for stroke recurrence, and the odds ratio (OR) and 95% CI were calculated for each variable.

### 2.3. Model building and validation

In our study, prediction models based on six different ML algorithms were used to analyze our data: linear regression algorithm (LR), plain Bayesian classification algorithm Naive Bayes model (NBC), decision tree algorithm (DT), random forest algorithm (RF), gradient augmentation algorithm (GBM), and xgboost (XGB) algorithm. Based on the training set data, average AUC values were calculated and the accuracy of the ML-based model algorithms was verified using the 10-fold crossover method. In addition, the ROC curves of various algorithmic models under the test set were plotted for external validation, while radar plots characterizing sensitivity, accuracy, and specificity of the models were provided to comprehensively evaluate performance of the models. The algorithm presenting the highest average AUC value was selected as the optimal algorithm. Then, contribution of each variable in the optimal model were calculated by the interpretable model SHAP to determine importance of the variables and the positive or negative contribution to the model. Finally, a web calculator was built on this basis to enable input of patient data and disease prediction to help physicians assess the risk of stroke recurrence within 1 year.

## 3. Results

### 3.1. Baseline population characteristics

**FIGURE 1:**
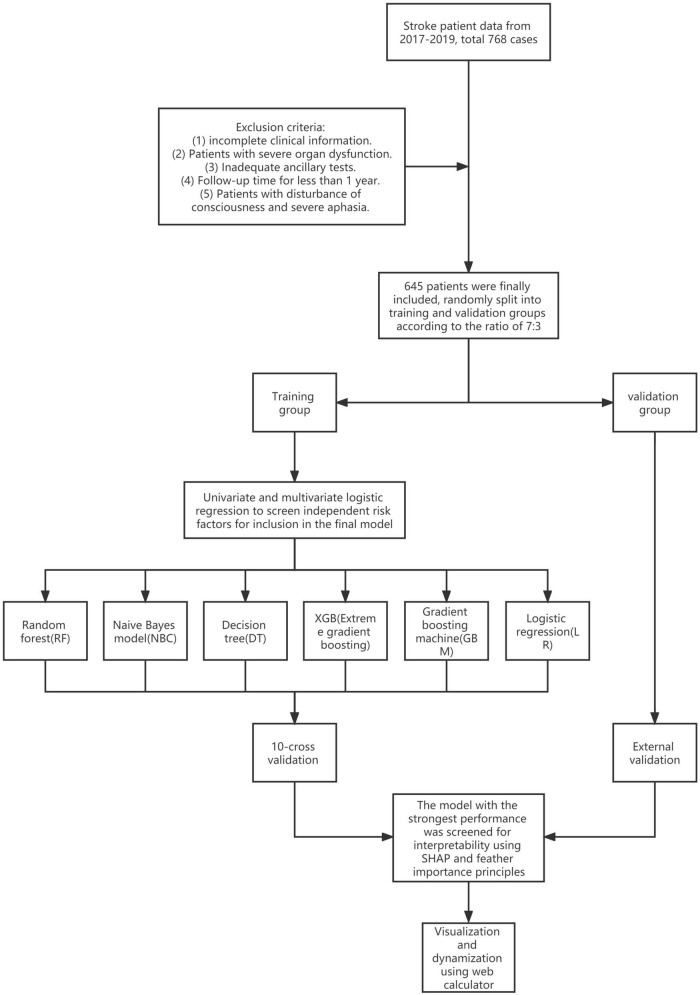
Flowchart of the whole experiment.

A total of 832 patients with AIS were included in this study, and 48 patients with incomplete clinical data and inadequate ancillary tests, 64 patients with disturbance of consciousness and severe aphasia, 18 patients with severe abnormal organ function, and 57 patients with less than 1 year of follow-up for various reasons were excluded. The final 645 patients with AIS with or without stroke recurrence within 1 year were included (Figure 1). A total of 84 patients experienced recurrent stroke. The rate of stroke recurrence was 13%. Table 1 shows that differences in the side of hemisphere (SOH), HCY, CRP, NSE, S100β, anticoagulation, PPI, dysphagia, and SS were statistically significant in the presence or absence of stroke recurrence. It is suggested that these variables may be independent risk factors for stroke recurrence.

**TABLE 1:** Baseline demographic and clinicopathological characteristics of all patients (stroke recurrence group and non-stroke recurrence group).

Characteristics	Level	Overall (*N* = 645)	No (*N* = 561)	Yes (*N* = 84)	*P*
Age, *n* (%)	< 60	362 (56.1)	311 (55.4)	51 (60.7)	0.429
	≥ 60	283 (43.9)	250 (44.6)	33 (39.3)	
Gender, *n* (%)	Female	263 (40.8)	233 (41.5)	30 (35.7)	0.372
	Male	382 (59.2)	328 (58.5)	54 (64.3)	
SBP, median (Q1, Q3)	NA	143.0 (132.0, 156.0)	143.0 (132.0, 156.0)	145.0 (132.0, 157.0)	0.705
DBP, median (Q1, Q3)	NA	87.0 (74.0, 97.0)	86.0 (74.0, 97.0)	88.0 (75.8, 96.0)	0.466
SD, *n* (%)	Anterior circulation	258 (40.0)	220 (39.2)	38 (45.2)	0.482
	Posterior circulation	235 (36.4)	209 (37.3)	26 (31.0)	
	Anterior/Posterior circulation	152 (23.6)	132 (23.5)	20 (23.8)	
SOH, *n* (%)	Left	271 (42.0)	228 (40.6)	43 (51.2)	0.014
	Right	256 (39.7)	221 (39.4)	35 (41.7)	
	Bilateral	118 (18.3)	112 (20.0)	6 (7.1)	
SOS, *n* (%)	Cortex	149 (23.1)	131 (23.4)	18 (21.4)	0.716
	Cortex-subcortex	147 (22.8)	123 (21.9)	24 (28.6)	
	Subcortex	176 (27.3)	155 (27.6)	21 (25.0)	
	Brainstem	100 (15.5)	89 (15.9)	11 (13.1)	
	Cerebellum	73 (11.3)	63 (11.2)	10 (11.9)	
NOS, *n* (%)	Single stroke lesion	453 (70.2)	390 (69.5)	63 (75.0)	0.37
	Multiple stroke lesions	192 (29.8)	171 (30.5)	21 (25.0)	
Cholesterol, median (Q1, Q3)	NA	5.3 (4.4, 6.2)	5.2 (4.4, 6.2)	5.6 (4.8, 6.0)	0.267
Triglyceride, median (Q1, Q3)	NA	2.2 (1.9, 2.4)	2.2 (1.9, 2.4)	2.2 (2.1, 2.4)	0.059
LDL, median (Q1, Q3)	NA	4.8 (4.3, 4.9)	4.7 (4.3, 5.0)	4.8 (4.4, 4.9)	0.772
FBG, median (Q1, Q3)	NA	5.2 (4.6, 5.8)	5.2 (4.6, 5.8)	5.3 (4.6, 6.0)	0.388
HBALC, median (Q1, Q3)	NA	5.6 (5.3, 5.9)	5.6 (5.3, 5.9)	5.7 (5.3, 6.0)	0.364
HCY, median (Q1, Q3)	NA	15.5 (12.6, 19.1)	14.7 (12.3, 18.0)	20.7 (18.7, 22.0)	< 0.001
UA, median (Q1, Q3)	NA	350.1 (310.8, 407.6)	350.1 (311.3, 406.8)	349.7 (307.5, 411.9)	0.809
MB, median (Q1, Q3)	NA	97.0 (74.9, 144.7)	97.7 (75.3, 146.7)	94.9 (71.9, 143.4)	0.58
CRP, median (Q1, Q3)	NA	11.9 (7.5, 17.1)	11.4 (7.3, 16.6)	14.9 (9.3, 20.1)	< 0.001
FIB, median (Q1, Q3)	NA	4.4 (4.0, 4.8)	4.3 (4.0, 4.7)	4.4 (4.0, 4.8)	0.449
D-dimer, median (Q1, Q3)	NA	174.0 (133.0, 221.0)	175.0 (134.0, 222.0)	158.5 (126.8, 201.5)	0.31
BNP, median (Q1, Q3)	NA	93.0 (73.0, 162.0)	93.0 (74.0, 161.0)	86.0 (69.8, 168.5)	0.792
NSE, median (Q1, Q3)	NA	16.2 (12.7, 18.6)	15.9 (12.6, 18.4)	17.7 (15.0, 19.0)	0.002
S100β, median (Q1, Q3)	NA	275.0 (223.0, 289.0)	274.0 (223.0, 288.0)	283.5 (241.5, 306.0)	0.012
Thrombolysis, *n* (%)	No	448 (69.5)	393 (70.1)	55 (65.5)	0.47
	Yes	197 (30.5)	168 (29.9)	29 (34.5)	
Thrombectomy, *n* (%)	No	614 (95.2)	535 (95.4)	79 (94.0)	0.584
	Yes	31 (4.8)	26 (4.6)	5 (6.0)	
Antiplatelet, *n* (%)	No	117 (18.1)	100 (17.8)	17 (20.2)	0.701
	Yes	528 (81.9)	461 (82.2)	67 (79.8)	
Anticoagulation, *n* (%)	No	553 (85.7)	495 (88.2)	58 (69.0)	< 0.001
	Yes	92 (14.3)	66 (11.8)	26 (31.0)	
Statin, *n* (%)	No	98 (15.2)	88 (15.7)	10 (11.9)	0.461
	Yes	547 (84.8)	473 (84.3)	74 (88.1)	
PPI, *n* (%)	No	519 (80.5)	461 (82.2)	58 (69.0)	0.007
	Yes	126 (19.5)	100 (17.8)	26 (31.0)	
Dysphagia, *n* (%)	No	525 (81.4)	464 (82.7)	61 (72.6)	0.039
	Yes	120 (18.6)	97 (17.3)	23 (27.4)	
SS, *n* (%)	No	380 (58.9)	356 (63.5)	24 (28.6)	< 0.001
	Yes	265 (41.1)	205 (36.5)	60 (71.4)	
SAP, *n* (%)	No	494 (76.6)	435 (77.5)	59 (70.2)	0.182
	Yes	151 (23.4)	126 (22.5)	25 (29.8)	

NA, not available; SD, stroke distribution; SOH, side of hemisphere; NOS, number of stroke lesions; SOS, site of stroke lesions; LDL, low-density lipoprotein; FBG, fasting blood glucose; HCY, homocysteine; UA, uric acid; FIB, fibrinogen; MB, myoglobin; CRP, C-reactive protein; BNP, brain natriuretic peptide; NSE, neuron-specific enolase; PPI, proton pump inhibitor therapy; SS, stroke severity, SAP, stroke-associated pneumonia.

**TABLE 2:** Univariate and multivariate logistic regression analysis of patients with recurrent stroke.

Characteristics	Univariate logistic analysis				Multivariate logistic analysis		
	OR	95% CI	*P*-value		OR	95% CI	*P*-value
**Age**							
< 60		Reference	Reference				
≥ 60	0.81	(0.50–1.28)	0.367				
**Gender**							
Female		Reference	Reference				
Male	1.28	(0.80–2.08)	0.315				
SBP	1	(0.99–1.01)	0.99				
DBP	1.01	(0.99–1.02)	0.522				
**SD**							
Anterior circulation		Reference	Reference				
Posterior circulation	0.72	(0.42–1.23)	0.23				
Anterior/posterior circulation	0.88	(0.48–1.56)	0.668				
**SOH**							
Left		Reference	Reference			Reference	Reference
Right	0.84	(0.52–1.36)	0.482		0.62	(0.33–1.15)	0.129
Bilateral	0.29	(0.11–0.66)	0.002		0.25	(0.09–0.73)	0.011
**SOS**							
Cortex		Reference	Reference				
Cortex-subcortex	1.42	(0.73–2.78)	0.302				
Subcortex	0.98	(0.50–1.95)	0.965				
Brainstem	0.9	(0.39–1.99)	0.806				
Cerebellum	1.16	(0.49–2.64)	0.728				
**NOS**							
Single stroke lesion		Reference	Reference				
Multiple stroke lesions	0.76	(0.44–1.27)	0.309				
Cholesterol	1.08	(0.91–1.29)	0.381				
Triglyceride	1.69	(0.93–3.08)	0.086				
LDL	1.1	(0.80–1.52)	0.544				
FBG	1.06	(0.84–1.35)	0.605				
HBALC	1.23	(0.70–2.16)	0.476				
HCY	1.57	(1.42–1.73)	< 0.001		1.6	(1.44–1.79)	< 0.001
UA	1	(1.00–1.00)	0.687				
MB	1	(1.00–1.00)	0.655				
CRP	1.07	(1.04–1.11)	< 0.001		1.06	(1.01–1.11)	0.012
FIB	1.1	(0.76–1.58)	0.622				
D-dimer	1	(1.00–1.00)	0.767				
BNP	1	(1.00–1.00)	0.476				
NSE	1.1	(1.03–1.17)	0.005		0.98	(0.88–1.09)	0.704
S100β	1.01	(1.00–1.01)	0.016		1	(1–1.01)	0.379
**Thrombolysis**							
No		Reference	Reference				
Yes	1.24	(0.75–2.00)	0.398				
**Thrombectomy**							
No		Reference	Reference				
Yes	1.33	(0.43–3.32)	0.583				
**Antiplatelet**							
No		Reference	Reference				
Yes	0.85	(0.49–1.56)	0.585				
**Anticoagulation**							
No		Reference	Reference			Reference	Reference
Yes	3.36	(1.96–5.68)	< 0.001		1.9	(0.9–4.02)	0.094
**Statin**							
No		Reference	Reference				
Yes	1.36	(0.70–2.91)	0.377				
**PPI**							
No		Reference	Reference			Reference	Reference
Yes	2.07	(1.22–3.42)	0.007		0.81	(0.37–1.78)	0.598
**Dysphagia**							
No		Reference	Reference			Reference	Reference
Yes	1.81	(1.05–3.03)	0.034		0.9	(0.41–1.97)	0.789
**SS**							
No		Reference	Reference			Reference	Reference
Yes	4.32	(2.64–7.27)	< 0.001		3.98	(1.98–7.97)	< 0.001
**SAP**							
No		Reference	Reference				
Yes	1.47	(0.87–2.42)	0.149	1.47			

### 3.2. Univariate and multivariate logistic regression

Univariate logistics regression analysis showed that right hemisphere, HCY, CRP, NSE, S100β, Anticoagulation, PPI, dysphagia, and SS all had statistically significant correlation with the occurrence of stroke recurrence. The nine variables mentioned above had *P* < 0.05 after univariate analysis were included in a multivariate logistics regression analysis, suggesting that right hemisphere, HCY, CRP, and SS, might be independent risk factors for stroke recurrence. The differences were all statistically significant [right (OR: 0.25, 95% CI 0.09–0.73, *P* < 0.05), HCY (1.6, 1.44–1.79, *P* < 0.05), CRP (1.06, 1.01–1.11, *P* < 0.05) and SS (3.98, 1.98–7.97, *P* < 0.001)].

### 3.3. Model building and performance validation

**FIGURE 2:**
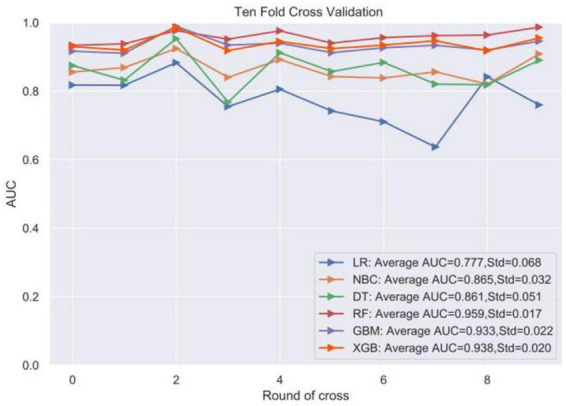
Ten-fold cross-validation within the training set of the machine algorithm.

Four significantly different factors were defined as variables of the model by single-factor and multi-factor screening, and the model was initially constructed by randomly slicing original data set into a training set and a test set with the ratio of 7:3, with 452 patients in training set and 193 patients in test set. Six ML algorithms including linear regression algorithm (LR), plain Bayesian classification algorithm (NBC), decision tree algorithm (DT), random forest algorithm (RF), GBM, and xgboost (XGB) algorithm were executed in the training set to build a prediction model. To avoid training chance errors, the performance of the models in fitting the original data was measured, visually compared. To improve the model prediction effectiveness, a 10-fold cross-validation method was used for internal validation (Figure 2). The results showed that the RF model was the best predictor of stroke recurrence (mean AUC 0.959, standard deviation 0.017) and the LR model had the lowest AUC value of 0.777.

**FIGURE 3:**
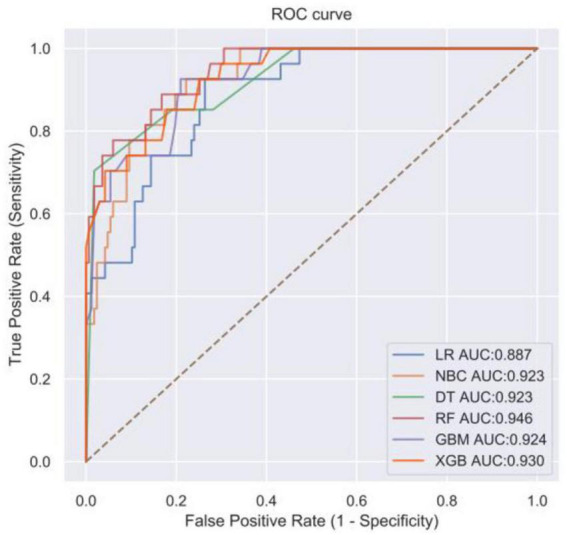
Receiver operating characteristic (ROC) curve of machine algorithm model under the test set.

**FIGURE 4:**
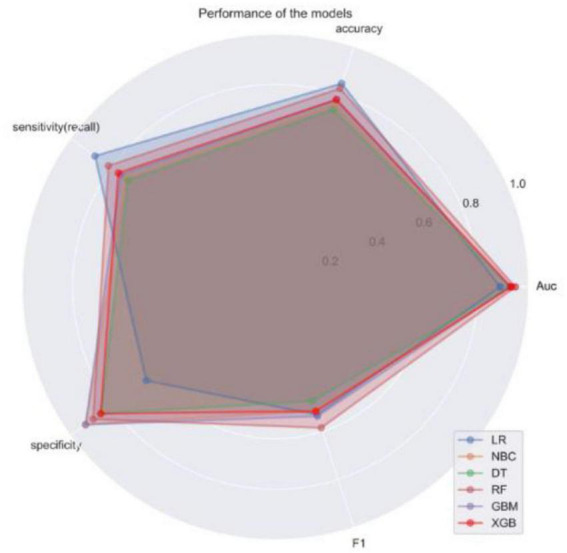
Radar plots of sensitivity and specificity of 6 machine algorithm models.

In addition, the ROC curves of various algorithmic models under the test set (Figure 3) were plotted for external validation in this study, where the RF algorithm prediction model had the largest area under the AUC curve (AUC = 0.946), the LR algorithm prediction model had the lowest (AUC = 0.887), and the other models were in between, indicating that the RF model had a better performance in terms of data fitting effect. The radar plot of prediction model sensitivity and specificity showed (Figure 4) that the LR and RF models had better accuracy and sensitivity in their prediction ability, while the NBC model had higher specificity. However, the RF model approach was more effective (F1 = 0.585) when considering the precision and recall rates (F1) together. Finally, the performance of the six algorithmic models was compiled into a table (Table 3). Therefore, we chose the RF model as the final prediction model.

**TABLE 3:** Summary of specific performance of six machine algorithm models.

Scoring	LR	NBC	DT	RF	GBM	XGB
AUC	0.887	0.923	0.923	0.946	0.924	0.930
accuracy	0.845	0.758	0.737	0.825	0.778	0.778
Sensitivity (recall)	0.880	0.731	0.719	0.814	0.754	0.766
specificity	0.630	0.926	0.852	0.889	0.926	0.852
F1	0.531	0.515	0.474	0.585	0.538	0.517

### 3.4. Interpretability study of variables in the RF model

**FIGURE 5:**
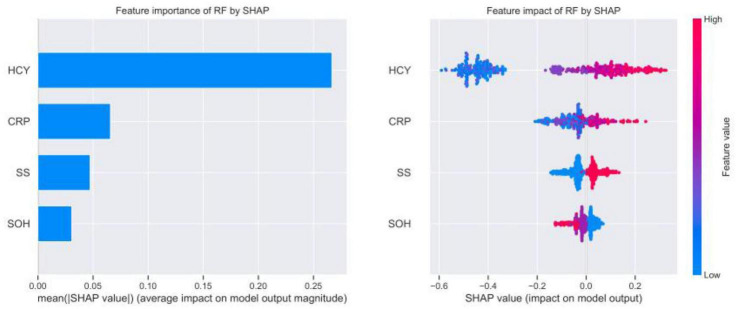
Relative importance of variables based on SHAP for RF prediction model.

Considering clinical utility, we focused on the contribution of each variable to the final prediction outcome in the ML-based RF prediction model for AIS patients using the interpretable model SHAP. With each variable as a participant and the model output as a collaborative outcome, the contribution, or SHAP value, was calculated. As can be seen in Figure 5 (left), HCY, CRP, SS, and SOH are in descending order. Meanwhile, Figure 5 (right) shows the magnitude of the four variables taking values in different colors, with higher values corresponding to red and vice versa. The figure takes the SHAP value of zero as the origin, and the negative left and positive right indicate that the variable contributes negatively or positively to the output prediction results. Therefore, we conclude that HCY has the greatest impact on the model, HCY, SS, and CRP all contribute positively to the RF model output prediction results, and SOH contributes negatively to the RF model output prediction results.

### 3.5. Web-based calculator RF model

**FIGURE 6:**
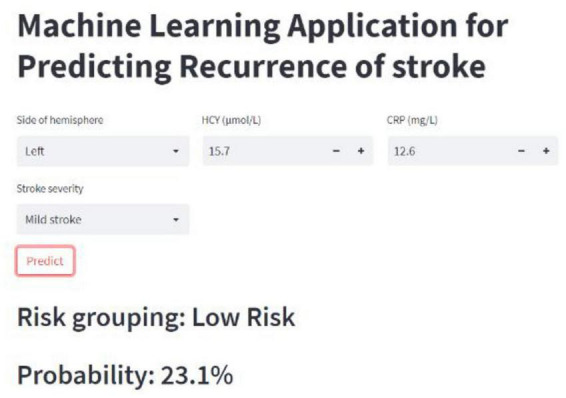
Online calculator for predicting stroke recurrence.

The RF-based model performed best among the six models. Therefore, we built a web-based calculator^1^ to facilitate the clinical application of this predictive model (Figure 6).

## 4. Discussion

Stroke prevalence is universal, but it is more likely to occur in the middle-aged and elderly population with higher rates of disability and mortality. Most strokes are predominantly ischemic strokes caused by arterial occlusion, and stroke recurrence leads to a higher risk of death and disability than their first occurrence (Tu et al., 2021, 2022). Therefore, there is an urgent need to identify risk factors for stroke recurrence in such patients to improve prevention, reduce recurrence and disability rates, and prolong patient survival. Traditional data mining and statistical methods usually require feature engineering to obtain effective and more robust features, and then construct prediction or clustering models. With complex data and a lack of sufficient domain knowledge, both steps present many challenges (Miotto et al., 2018). Machine learning utilizes large-scale, diverse datasets to build useful patterns by running complex algorithms, and has an important role in the biomedical field for disease detection, diagnosis, prevention, and treatment. Its development leads to more accurate early diagnosis, individualized treatment, and continuous monitoring, as well as effective screening for disease-related risk factors and prediction of disease recurrence (Goecks et al., 2020). Machine learning is particularly useful when datasets are too large or complex for human analysis, and/or when you want to automate the data analysis process to build reproducible and time-saving pipelines. The RF model used in the study, for example, has the advantage of knowing how important each element is to the prediction, and the individual DTs are human-readable, making them easier to train and adjust. But there are also shortcomings that are not suitable for regression and many DTs are difficult to explain (Greener et al., 2022).

Throughout the study, we followed 645 patients with AIS, 84 of whom experienced recurrence. The rate of stroke recurrence was consistent with previous result (13% vs. 11.1%) (Hankey, 2014). After evaluating baseline characteristics of multiple clinical variables collected with stroke recurrence and identifying four independent risk factors for stroke recurrence by univariate and multivariate LR analysis (i.e., right hemisphere, HCY, CRP, and SS), six ML methods were used: LR, NBC, DT, XGB, GBM, and RF. They were used to develop prediction models for individualized prediction of stroke recurrence in AIS patients (Figure 1). The results showed that the RF model had the best predictive ability, and the mean AUC of all 10-fold cross-validation results were > 0.8. After a comprehensive evaluation of the performance of the six models (Table 3), it was concluded that the RF model performed better, and the relative importance of the four risk variables in the RF model was compared from highest to lowest, HCY, CRP, SS, and SOH. Finally, an online web calculator was created to facilitate clinical application.

Table 1 shows the baseline characteristics of patients with AIS. There were no significant differences between non-stroke recurrent patients and stroke recurrent patients in terms of gender and age; however, there were significant differences in the side of the hemisphere (SOH; *P* = 0.014 < 0.05), blood homocysteine (HCY; *P* < 0.0 01), C-reactive protein (CRP; *P* < 0.001), neuron-specific enolase (NSE; *P* = 0.002 < 0.05), central neurospecific protein (S100β; *P* = 0.012 < 0.05), anticoagulation therapy (*P* < 0.001), proton pump inhibitor therapy (PPI; *P* = 0.007 < 0.05), dysphagia (*P* = 0.039 < 0.05), and stroke severity (SS; *P* < 0.001).

HCY, CRP, NSE, and S100β are all serum proteins. HCY is a sulfur-containing amino acid in the body and an important intermediate in the metabolism of methionine and cysteine, which itself is not involved in protein synthesis. Elevated HCY has become an independent risk factor for the development of atherosclerosis. As a pro-inflammatory marker, the inflammatory process has an important role in pathophysiology of ischemic stroke, and elevation of HCY is considered a risk factor for ischemic stroke (Chen et al., 2017). The data in Table 1 show that HCY was lower in patients with recurrent stroke than in patients with non-recurrent stroke (13.2 vs. 16.2, *P* = 0.002). Previously, it was shown that hypertension with high homocysteine (HHcy) (H-type hypertension) and CRP can increase the incidence of ischemic stroke. Later data demonstrated that recurrent ischemic stroke (RIS) is associated with advanced age, male sex, diabetes, H-type hypertension, and C-reactive protein. In contrast, controlling H-type hypertension and C-reactive protein levels reduce the risk of RIS (Zhang et al., 2016). Under normal conditions, the levels of NSE and S100β in body fluids are extremely low. When neuronal injury or necrosis occurs, NSE and S100β rapidly spill from the cells into the cerebrospinal fluid and enter the blood through the damaged blood-brain barrier, resulting in elevated serum NSE and S100β concentrations. The levels of which reflect the extent of neuronal damage, so elevated NSE and S100β suggest possible relapse. In addition, other serum biomarkers, such as serum Copeptin levels, are associated with recurrent stroke events and are predictors of severity at admission and 1-year stroke recurrence in stroke patients (Tang et al., 2017). In addition, other biomarkers such as serum fatty acid binding protein 4 (FABP4) (Li et al., 2019), serum CXCL12 levels (Gu et al., 2016), interleukin-37 (Zhang et al., 2021), and cystatin C (Liu et al., 2021) have been reported to be associated with stroke recurrence.

Serum C-reactive protein, an acute chronotropic reactive protein elevated in the presence of infection, is second only to HCY in RF models in terms of relative importance. It is also a non-specific marker of systemic inflammation reflecting various infectious and non-infectious inflammatory conditions in the organism. A retrospective review showed that 26 studies reported an association of CRP with recurrent stroke, of which 12 (46%) described a positive association (McCabe et al., 2021), a result that is consistent with what we obtained. In addition to the above-mentioned control of CRP with H hypertension that reduces the risk of RIS, elevated serum Hs-CRP and HCY levels are associated with the risk of post-stroke depression (PSD) 1 year after stroke onset, and the combination of these two factors adds prognostic information to early assessment of PSD (Cheng et al., 2018).

The study in Table 2 found that the differences in NSE, S100β, Anticoagulation, PPI, and dysphagia were statistically significant in the univariate LR analysis, but the differences were not found to be statistically significant when the above variables were included in the multivariate LR analysis, which may be due to the sample size of the study population, selection bias. SS was a good independent predictor of stroke recurrence (Table 2), with a risk ratio of 3.98 for recurrence in stroke patients, as well as being a relatively important factor in the RF model. In a 2016 study analyzing the regression after ischemic stroke and its associated factors in elderly patients, it was shown that at 12 months of stroke, moderate stroke was associated with dependency and severe stroke was associated with dependency and recurrence (Wu et al., 2016). In addition, a meta-analysis of stroke recurrence rates was recently performed in a retrospective study of patients with first ischemic stroke. The results of this study showed that hypertension, diabetes mellitus, atrial fibrillation, transient ischemic attack, and high SS were independent risk factors for recurrence (Kolmos et al., 2021).

The right hemisphere is the last relative importance in the RF model. Stroke patients experience impairments such as contralateral motor deficits and interhemispheric imbalances including hyperexcitability of the contralateral hemisphere after stroke. Since the recovery of cerebral hemispheres through motor dysfunction can be achieved by increasing excitability of the affected hemisphere or decreasing the excitability of the unaffected hemisphere, current brain treatments for stroke patients include a brain-computer interface (BCI) and transcranial magnetic stimulation (TMS) therapies to reduce mortality and alleviate the degree of disability in patients. Studies have shown that bilateral hemisphere treatment by TMS facilitates motor recovery of paralyzed hands in stroke patients (Takeuchi et al., 2009). In contrast, when patients present with bilateral focal hemispheres, there may be an interruption of the axis between the central nervous system and the gastrointestinal system, leading to secondary symptoms such as dysphagia and gastrointestinal bleeding (Schaller et al., 2006). Similarly, our study data show that dysphagia, although not an independent risk factor for stroke recurrence, has a statistically significant difference between the baseline characteristics of patients with and without stroke recurrence. Importance of primary prevention in patients with the first stroke and secondary prevention in recurrent stroke is stressed in the current study. Primary prevention treatment is anticoagulation for atrial fibrillation, antihypertensive treatment for hypertension and controlling glucose for diabete, etc (Diener and Hankey, 2020). The primary aim of secondary prevention is to prevent or reduce the risk of recurrent stroke and to reduce the degree of disability. Effective treatments include antithrombotic and anticoagulant therapy, revascularization, and implementation of structured evaluation and intervention (Hankey, 2014). Although effective for secondary prevention of ischemic stroke with aspirin, increases the risk of hemorrhagic stroke, upper gastrointestinal bleeding (UGIB), and dyspepsia. Prophylactic administration of proton pump inhibitors (PPIs) may reduce the risk of these digestive symptoms (Takabayashi et al., 2015). There is evidence that some proton pump inhibitors can attenuate the antiplatelet effects of clopidogrel, but after multivariate adjustment, the data show that the use of proton pump inhibitors is not associated with a significantly increased risk of recurrent stroke or death (Juurlink et al., 2011). Again, this is consistent with the conclusions reached in this study.

However, there are some limitations that need to be addressed in the future. First, the ML algorithm model we developed is limited to one hospital, which may limit its widespread use in other regions. Second, the sample size of this study has some limitations and there is room for extending the follow-up period. Finally, this study is retrospective and suffers from the inherent data bias of retrospective studies, which may lead to bias in the data. We will conduct further multicenter and prospective studies in the future.

## 5. Conclusion

In conclusion, we constructed six risk prediction models for stroke recurrence in patients with AIS by machine learning algorithm (ML), introducing four independent risk factors associated with stroke recurrence (i.e., right hemisphere, HCY, CRP, and SS). Among them, we found that RF model made promising prediction, as it performed the best in both internal validation and external validation combined, with comparable accuracy, sensitivity, and specificity. It is hoped that this web-based calculator can serve as an effective predictive tool to help stroke patients prevent recurrence and assist physicians in adjudication.

## Data availability statement

The raw data supporting the conclusions of this article will be made available by the authors, without undue reservation.

## Ethics statement

The studies involving human participants were reviewed and approved by the Ethics Committee of The Second Affiliated Hospital of Xuzhou Medical University. The patients/participants provided their written informed consent to participate in this study.

## Author contributions

WlL, LQR, and XW completed the study design. KW, WcL, and WlL performed the study and collected and analyzed the data. QS, CaS, and WlL drafted the manuscript. LQR, XW, KW, and HL provided the expert consultations and suggestions. CX and CY conceived of the study, participated in its design and coordination, and helped to embellish language. All authors reviewed the final version of the manuscript and approved the submitted version.

## Footnotes

1
https://www.r-project.org/
