# Corrigendum: A machine learning model for visualization and dynamic clinical prediction of stroke recurrence in acute ischemic stroke patients: a real-world retrospective study

**DOI:** 10.3389/fnins.2023.1235340

**Published:** 2023-07-10

**Authors:** Kai Wang, Qianqian Shi, Chao Sun, Wencai Liu, Vicky Yau, Chan Xu, Haiyan Liu, Chenyu Sun, Chengliang Yin, Xiu'e Wei, Wenle Li, Liangqun Rong

**Affiliations:** ^1^Department of Neurology, The Second Affiliated Hospital of Xuzhou Medical University, Xuzhou, Jiangsu, China; ^2^Key Laboratory of Neurological Diseases, The Second Affiliated Hospital of Xuzhou Medical University, Xuzhou, Jiangsu, China; ^3^State Key Laboratory of Molecular Vaccinology and Molecular Diagnostics & Center for Molecular Imaging and Translational Medicine, School of Public Health, Xiamen University, Xiamen, China; ^4^Department of Neurosurgery, The Second Affiliated Hospital of Soochow University, Suzhou, China; ^5^Department of Orthopaedic Surgery, The First Affiliated Hospital of Nanchang University, Nanchang, China; ^6^Division of Oral and Maxillofacial Surgery, Columbia University Irving Medical Center, New York, NY, United States; ^7^Department of Dermatology, Xianyang Central Hospital, Xianyang, China; ^8^Faculty of Medicine, Macau University of Science and Technology, Macau, China

**Keywords:** stroke, recurrence, machine learning, SHAP, web calculator

In the published article, there was an error in the legend for Figures 2–6 as published. Due to the unfamiliarity of some graduate students in our team with the submission system and the operation process of the writing software, the final version of the image was incorrectly uploaded as the image in the middle of the iteration of our machine learning algorithm model, not the final result of the model. The corrected legend appears below.

**Figure 2:**
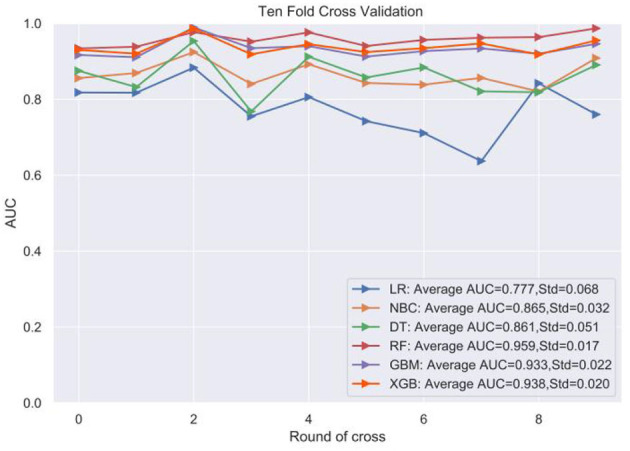
Ten-fold cross-validation within the training set of the machine algorithm.

**Figure 3:**
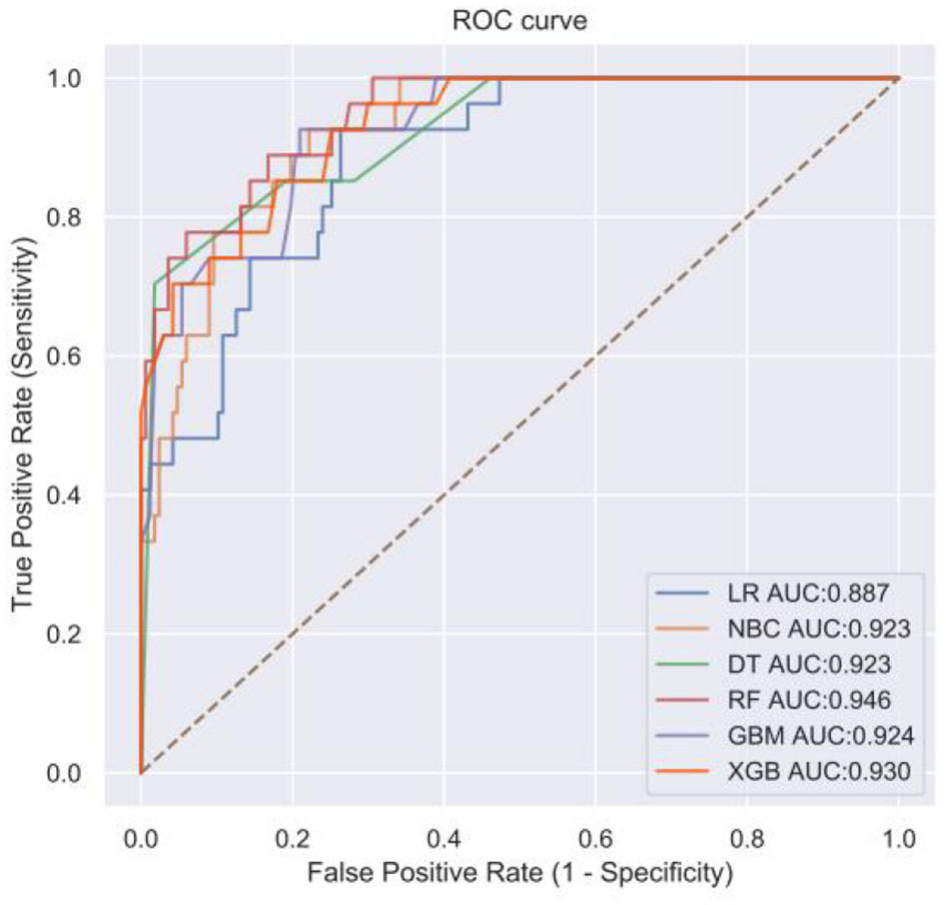
Receiver operating characteristic (ROC) curve of machine algorithm model under the test set.

**Figure 4:**
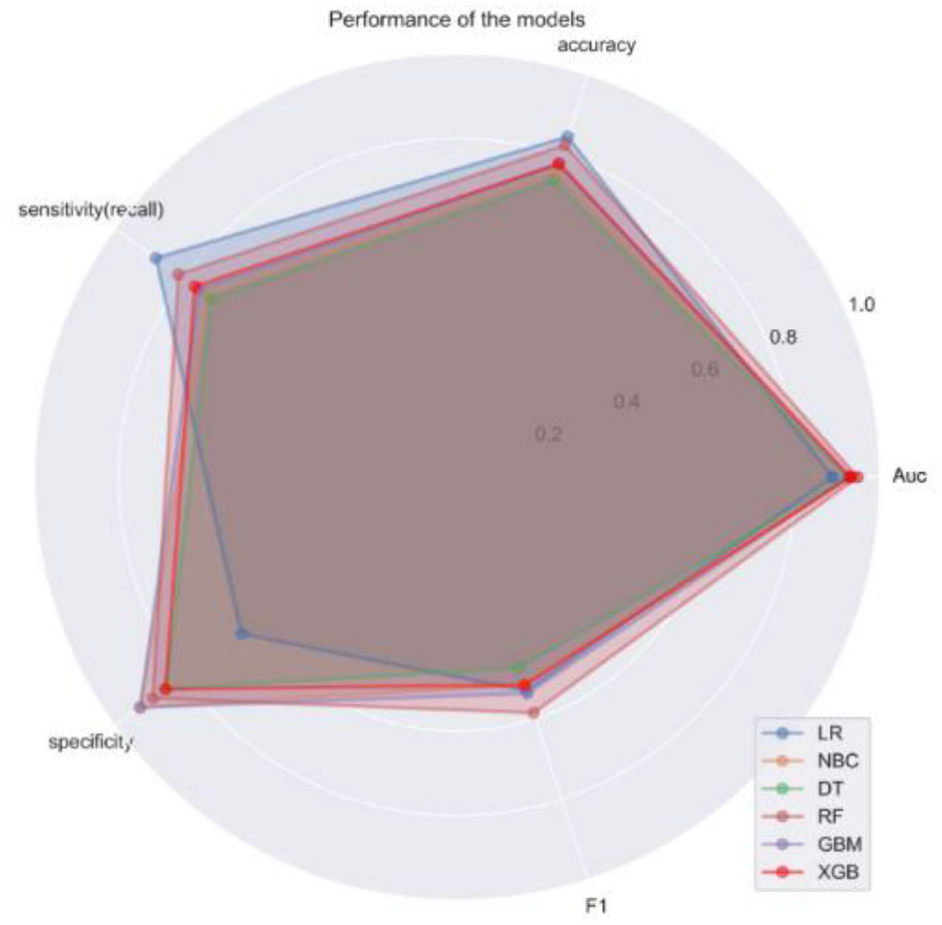
Radar plots of sensitivity and specificity of 6 machine algorithm models.

**Figure 5:**
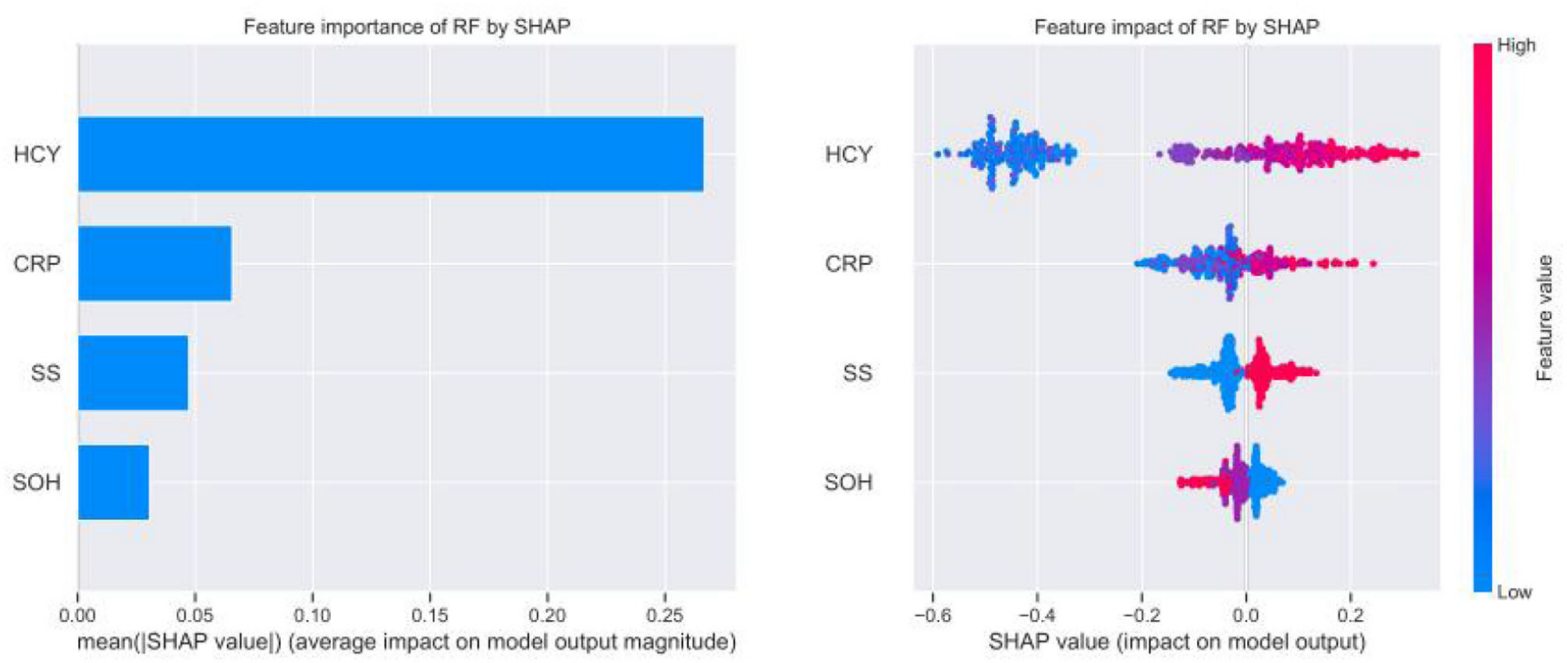
Relative importance of variables based on SHAP for RF prediction model.

**Figure 6:**
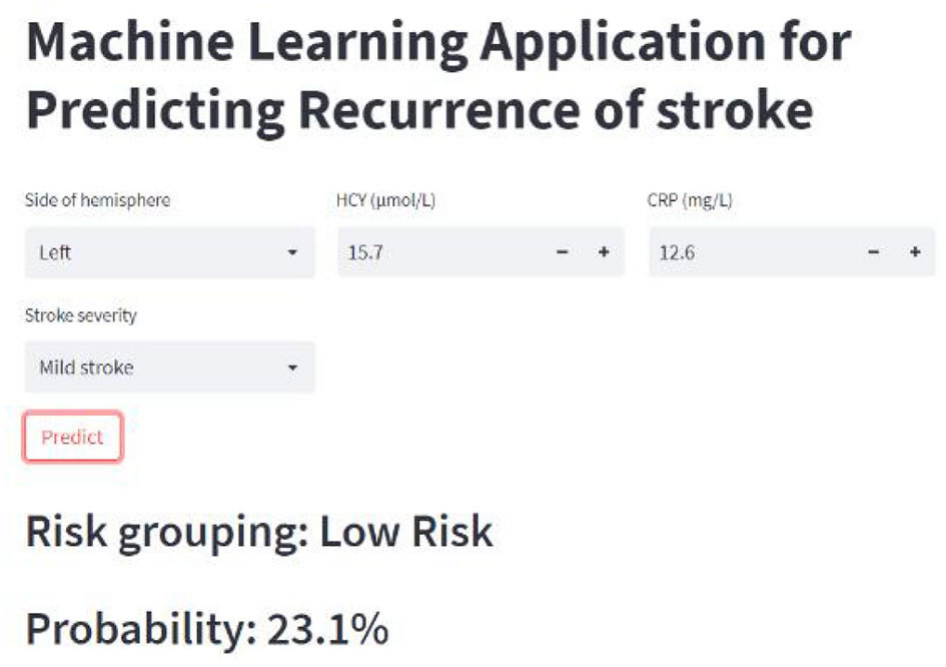
Online calculator for predicting stroke recurrence.

The authors apologize for these errors and state that this does not change the scientific conclusions of the article in any way. The original article has been updated.

